# Identification of QTLs and allelic effect controlling lignan content in sesame (*Sesamum indicum* L.) using QTL-seq approach

**DOI:** 10.3389/fgene.2023.1289793

**Published:** 2023-12-11

**Authors:** Sungup Kim, Eunsoo Lee, Jeongeun Lee, Yeon Ju An, Eunyoung Oh, Jung In Kim, Sang Woo Kim, Min Young Kim, Myoung Hee Lee, Kwang-Soo Cho

**Affiliations:** ^1^ Upland Crop Breeding Research Division, Department of Southern Area Crop Science, National Institute of Crop Science, Rural Development Administration, Miryang, Republic of Korea; ^2^ Central Crop Breeding Research Division, Department of Central Area Crop Science, National Institute of Crop Science, Rural Development Administration, Suwon, Republic of Korea

**Keywords:** DNA marker, lignan, oilseed, sesame, QTL-seq

## Abstract

Sesame (*Sesamum indicum* L.), an oilseed crop, is gaining worldwide recognition for its healthy functional ingredients as consumption increases. The content of lignans, known for their antioxidant and anti-inflammatory effects, is a key agronomic trait that determines the industrialization of sesame. However, the study of the genetics and physiology of lignans in sesame is challenging, as they are influenced by multiple genes and environmental factors, therefore, the understanding of gene function and synthetic pathways related to lignan in sesame is still limited. To address these knowledge gaps, we conducted genetic analyses using F7 recombinant inbred line (RIL) populations derived from Goenbaek and Gomazou as low and high lignin content variants, respectively. Using the QTL-seq approach, we identified three loci, *qLignan1-1*, *qLignan6-1*, and *qLignan11-1*, that control lignan content, specifically sesamin and sesamolin. The allelic effect between loci was evaluated using the RIL population. *qLignan6-1* had an additive effect that increased lignan content when combined with the other two loci, suggesting that it could be an important factor in gene pyramiding for the development of high-lignan varieties. This study not only highlights the value of sesame lignan, but also provides valuable insights for the development of high-lignan varieties through the use of DNA markers in breeding strategies. Overall, this research contributes to our understanding of the importance of sesame oil and facilitates progress in sesame breeding for improved lignan content.

## 1 Introduction

Sesame (*Sesamum indicum L.*) is a major oil crop and a special crop in East Asia, including Korea, China, and Japan, with a focus on its functional ingredients (Langyan et al., 2022). With an oil content of approximately 50% within its seeds (Uzun et al., 2008; Wang et al., 2014; Wei et al., 2015), sesame is an optimal candidate for industrial oil production and use. Extensive research has highlighted the nutritional advantages and health effects of sesame oil in humans, which include anti-inflammatory and anti-cancer activity, antioxidant properties, and nootropic effects (Yokota et al., 2007; Monteiro et al., 2014; Wan et al., 2015; Xu et al., 2015; Kim et al., 2023). Lignans, predominantly sesame oil, are categorized into fat-soluble and water-soluble types (Dar and Arumugam, 2013). Among these, sesamin and sesamolin are the major components of the fat-soluble aglycons (Dar and Arumugam, 2013; Michailidis et al., 2019), demonstrating the substantial health-promoting potential of sesame both clinically and pharmacologically (Majdalawieh et al., 2017). Given their significance, in-depth exploration of functional elements such as oil and lignans may significantly elevate the agricultural value and industrial potential of sesame.

In recent years, major crop breeding strategies have changed significantly, moving from traditional breeding using physical markers to the concept of marker-assisted selection (MAS) employing contemporary molecular markers (Collard and Mackill, 2008; Nadeem et al., 2018). In addition, the availability of high quality reference genomes and pan-genomes has made a variety of genomic studies possible, such as genetic mapping, structural variant studies of whole genomes, and syntenic analyses that compare regions between and within species (Bayer et al., 2020; Khan et al., 2020; Sun et al., 2022). The progress related to DNA markers based on next-generation sequencing (NGS) technology has made it possible to use genome-based markers such as single nucleotide polymorphisms (SNPs) and insertion–deletions (InDels), which are higher in throughput than previous RLFPs, RAPDs, and SSRs. This has led to rapid and accurate breeding as well as detailed research at the gene level, giving rise to a paradigm of digital breeding (Collard and Mackill, 2008; Ray and Satya, 2014; Nadeem et al., 2018; Varshney et al., 2021).

QTL-seq is an analytical technique that detects significant genetic regions associated with a trait by pooling individuals with contrasting phenotypes and comparing their genotypes to identify quantitative trait loci (QTLs) (Takagi et al., 2013). This approach combines bulked-segregant analysis (BSA), which previously used a small number of markers, with whole genome sequencing (WGS) using NGS technology to develop and use a large number of markers at low cost, thus improving the efficiency and accuracy of genetic studies. Unlike traditional QTL analysis in a bi-parental population, where all individuals must be genotyped to detect QTLs, QTL-seq mixes individuals with the same phenotype into groups, assuming that all genetic regions are equally likely to segregate due to random crossover, and only identifies genetic regions that are extremely segregated between groups as statistically significant (Abe et al., 2012; Takagi et al., 2013; Sugihara et al., 2022). Several studies in rice, maize, and tomato have demonstrated that QTL-seq provides comparable reliable results, especially for genotyping, thus overcoming the high cost of conventional QTL and genome wide association study (GWAS) (Chen et al., 2021; Topcu et al., 2021; Yang et al., 2021). Thus, a strategy to find useful trait-associated loci from a large number of materials based on different resources from different environments and ecotypes using low-cost and time-saving methods such as QTL-seq to accelerate breeding is essential. Secondary metabolites, are particularly regarded as typical quantitative features and are known to be influenced by a variety of hereditary variables (Alseekh et al., 2015).

In this study, we aimed to find genetic factors controlling sesame lignan content by using QTL-seq on an F7 recombinant inbred lines (RILs)constructed from individuals with different lignan content. Additionally, we looked at the impact of QTL stacking by comparing lignan content according to the haplotypes in the identified QTLs, which will be used as a basis for breeding the selection of new sesame varieties with improved economic value and functional content.

## 2 Materials and methods

### 2.1 Plant materials and phenotypic evaluation

A total of 257 F7 populations were derived by crossing low-lignan Goenbaek with high-lignan Gomazou through generational advancement using the single-seed descent method. Goenbaek is an elite variety most widely grown in South Korea for blight resistance and agronomic stability (Kim et al., 2018), while Gomazou is a variety bred in Japan for functionality (Yasumoto and Katsuta, 2006). The parental lines and RILs were planted at the National Institute of Crop Science experimental greenhouse, Miryang, South Korea, in June 2018 at 55 cm × 15 cm spacing; ten individuals of each line were harvested in August, and these planting formats and periods are adapted to the weather conditions in Korea. After harvesting, they were pooled and examined for sesamin and sesamolin content using HPLC to estimate the total lignan content. The sample was extracted in methanol and the analytical conditions were as follows: mobile phase A was 0.1% trifluoroacetic acid in water mobile phase B was 0.1% trifluoroacetic acid in methanol, and analysis under 60% (v/v) methanol conditions. The column was a YMC Triart C-18 (1.9 µm, 2.0 mm × 50 mm, 30°C; YMC Co., Kyoto, Japan) with a flow rate of 0.3 mL/min and was analyzed under UV at 290 nm using a Dionex 3000 HPLC (Thermo Fisher Scientific, Waltham, MA, United States of America). Sesamin and sesamolin were measured using an analytical standard (Sigma-Aldrich, St. Louis, MO, United States of America).

### 2.2 Construction of bulks, DNA extraction, and sequencing

Based on the distribution of lignan content in the RIL population, ten individuals belonging to each of the two extremes were selected to form high and low bulk. Genomic DNA was extracted from fresh leaves collected at the early stage of growth using the GeneAll^®^ Exgene™ Plant SV kit (GeneAll Biotechnology, Seoul, Korea). The DNA concentration of each individual was uniformly diluted to 10 ng/mol using the Nanodrop 3,000 spectrometer (Thermo Scientific, Wilmington, DE, United States of America) and pooled into high and low bulk by mixing an equal amount of DNAs, respectively. After constructing the pair-end sequencing libraries, Goenbaek (read length: 101 bp), Gomazou (read length: 151 bp), high bulk (read length: 151 bp), and low bulk (read length: 151 bp) were sequenced using the Illumina HiSeq platform. Sequenced data were deposited into the National Center for Biotechnology Information (NCBI) under BioProject (https://www.ncbi.nlm.nih.gov/bioproject) as PRJNA1028140.

### 2.3 Genotyping and QTL-seq analysis

The raw reads were trimmed to remove residual adapter sequences using trimmomatic v0.39 (Bolger et al., 2014). The trimmed reads were aligned to the sesame reference genome (cv. Zhongzhi No.13. v 2.0) (Wang et al., 2016) using BWA v0.7.17 (Li and Durbin, 2009), and BAM files were generated with a Q-score of 30 or higher using SAMtools v.1.15.1 (Li et al., 2009). To select variants for analysis, only positions with a mapping quality ≥30 or more, a mapping depth ≥8 but <250, and where both parental lines were confirmed to be homozygous alleles were filtered from the bulk samples using VCFtools v0.1.16 (Danecek et al., 2011). For the QTL-seq study, the SNP-index was calculated according to well-established principles from previous studies (Abe et al., 2012; Takagi et al., 2013), and the analysis pipeline was applied using VCF files (Sugihara et al., 2022). To reduce the possibility of missing positions and to find major genetic regions, the variant index of both Goenbaek and Gomazou was calculated and compared as consensus genomes. The average SNP-index was calculated through sliding window analysis with a 1 Mb window size and a step size of 50 Kb. Under the null hypothesis of no QTL, the statistical test of △(SNP-index) was performed for all SNP positions based on read depth. The △(SNP-index) was plotted along the physical chromosome position with 99% and 95% confidence intervals.

### 2.4 Variant mining, KEGG pathway mapping, and qRT-PCR analysis

The parent sequence variation and two bulks were analyzed using the SnpEff software to assign their changes and impacts on the function of the gene (Cingolani et al., 2012). For functional annotation of the genes, homologous genes were searched through comparative analysis using the BLASTp v2.10.0+ software with default parameters based on *Arabidopsis thaliana* gene–protein information. The functional descriptions of sesame genes were inferred by referring to *A. thaliana* homologs, and protein sequences and gene information were downloaded from TAIR version 10 (https://www.arabidopsis.org). The assignment of candidate genes to metabolic pathways was mapped to the Kyoto Encyclopedia of Genes and Genomes (KEGG, https://www.genome.jp/kegg/pathway.html) pathway. To compare gene expression between the Goenbaek and Gomazou varieties, total RNA was extracted from immature seeds at 15 and 30 days after flowering using the GeneAll Ribospin™ Plant kit (GeneAll, Korea) and converted into cDNA using the RNA to cDNA EcoDry™ Premix (Oligo dT) kits (TaKaRa, Japan). Primers for qRT-PCR were designed using Primer3 (https://primer3plus.com) as shown in Supplementary Table S1. qRT-PCR was performed using SsoAdvanced™ Universal SYBR^®^ Green Supermix (Bio-Rad Laboratories, CA, United States of America) on QuantStudio™ 5 (Applied Biosystems, United States of America). The amplification conditions were as follows: 50°C for 2 min and denaturation at 95°C for 10 min, followed by 40 cycles of 95°C for 15 s and 60°C for 1 min, 72°C for 15 s. The sesame housekeeping gene 18s rRNA (qFw: 5’-CGT​CCC​TGC​CCT​TTG​TAC​AC-3’ and qRv: 5’-CGA​ACA​CTT​CAC​CGG​ACC​AT-3’) was used as a reference gene (Noguchi et al., 2008). The relative quantification of samples was calculated according to the 2^-△△^CT method (Livak and Schmittgen, 2001) in three biological replicates, each with two technical replicates. Student’s t-test was used to determine statistical significance between groups.

### 2.5 Development of KASP, InDel, and CAPS markers and evaluation in RILs

Based on variant annotation results between the parents, Indel, Kompetitive Allele Specific PCR (KASP), and Cleaved Amplified Polymorphic Sequences (CAPS) markers were designed using bulk-biased SNPs and InDels located in the intron of *qLignan1-1*, and in the CDS of *qLignan6-1*, and in the intergenic region of *qLignan11-1*. Primers for InDel marker were designed using Primer3 (https://primer3plus.com), while primers for CAPS marker were designed using dCAPS Finder 2.0 (http://helix.wustl.edu/dcaps). PCR amplification conditions were: 95°C for 5 min, followed by 30 cycles of 95°C for 10 s, 60°C for 15 s, and 72°C for 15 s. For CAPS marker assay, amplified products were digested with *BspHI* restriction enzymes. Amplified or digested products were separated and visualized using QIAxcel (an automated capillary electrophoresis system by Qiagen, Hilden, Germany) for genotyping. The KASP primer design was performed by LGC genomics (London, UK) for two allele-specific forward primers and a common reverse primer based on 100 bp of both flanking sequences. The KASP-PCR amplification was conducted following the KASP technology manual (LGC Genomics, Beverly, MA, United States of America): 94°C for 15 min of activation, 10 cycles of 94°C for 20 s and a gradual decrease from 61°C (decrease at 0.6°C per cycle), and 26 cycles of 94°C for 20 s and 55°C for 1 min.

## 3 Results

### 3.1 Sesamin, sesamolin, and lignan content in the parent and RIL, and the construction of two extreme bulks

**FIGURE 1:**
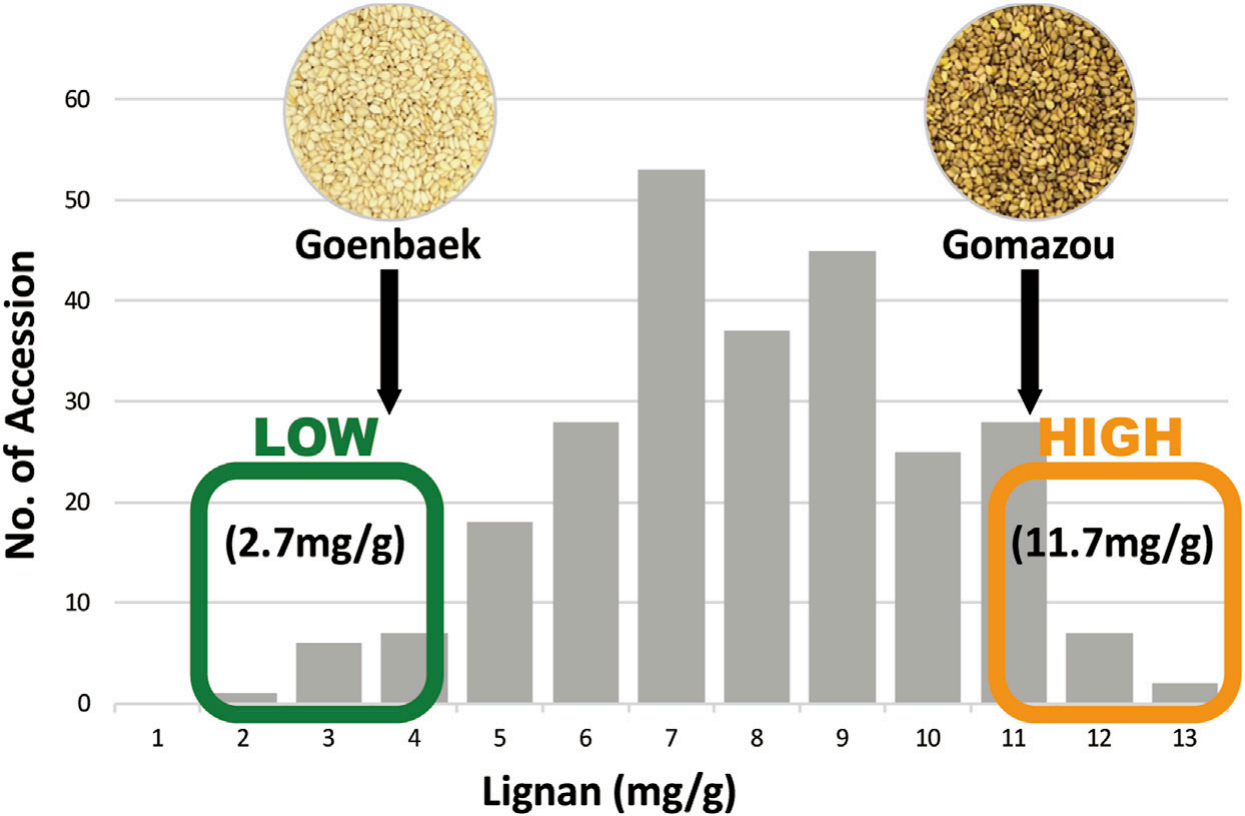
Frequency distribution of lignan content in the population of Goenbaek × Gomazou Recombinant Inbred Lines (RILs). Arrows indicate the mean lignin content of the parental variants. The average lignan content is represented in low and high bulk, respectively.

The sesamin and sesamolin content in the Goenbaek, Gomazou, and 257 F7 RILs seeds were investigated and the two compounds were combined to give the total lignan content. Goenbaek and Gomazou seeds had mean sesamin, sesamolin, and lignan contents of 2.7 and 8.2 mg/g, 1.0 and 3.1 mg/g, and 3.7 and 11.3 mg/g, respectively; Gomazou seeds had higher amounts of all components (Table 1). The 257 RIL accessions had mean sesamin, sesamolin, and lignan contents of 5.2, 2.2, and 7.5 mg/g, respectively, and the observed minimum and maximum within-population values suggested that some accessions resulted from transgressive segregation beyond the parental values (Table 1). Statistically significant correlations were observed between the three compounds (Supplementary Figure S1). In particular, sesamin (r = 0.98, *p* < 0.001) and sesamolin (r = 0.85, *p* < 0.001) showed strong positive correlations with lignan. Based on the normal distribution of lignan content in the population, 10 individuals were selected from each end of the extremes to comprise a low bulk (Lignan-L) ranging from 1.7 to 3.5 mg/g and a high bulk (Lignan-H) ranging from 10.8 to 12.8 mg/g, with a mean of 2.7 and 11.7 mg/g, respectively (Figure 1, Supplementary Table S2).

**TABLE 1:** Variation of sesamin, sesamolin, and lignan content in the parental lines and Recombinant Inbred Lines (RILs) population.

Lignan content (mg/g)	Parents		RIL population			
	Goenbaek	Gomazou	Average	SD	Min	Max
Sesamin	2.7	8.2	5.2	1.7	1.0	9.4
Sesamolin	1.0	3.1	2.2	0.6	0.7	3.7
Lignan	3.7	11.3	7.5	2.1	1.7	12.8

### 3.2 Whole genome resequencing of parents and lignan-high and lignan-low bulks

A total of four samples were sequenced, including parents, Lignan-H, and Lignan-L, generating a total of 80.4 Gb of raw data (Supplementary Table S3). After filtering by quality score and adapter trimming, 170,831,013 reads (101 bp in length) were mapped for Goenbaek and 216,367,131 reads (151 bp in length) for Gomazou at average depths of 51x and 103x, respectively. Similarly, Lignan-L and Lignan-H mapped 48,240,157 and 48,403,785 reads, respectively, with an average depth of approximately 21x. All samples covered more than 95% of the sesame reference genome in terms of bases for which at least one read was mapped. Sequenced reads from the parental lines were deposited in the National Center for Biotechnology Information (NCBI) database under the BioProject accession number PRJNA1028140.

### 3.3 Calculation of SNP/InDel-index and QTL-seq analysis

To perform the QTL-seq analysis, Goenbaek and Gomazou were used as consensus genomes with replacement variants for the reference genome. The variants for analysis were selected by ensuring that only alleles that are homozygous and polymorphic to parental align to one another, resulting in a total of 481,298 SNP/InDel variants (416,102 SNPs and 65,195 InDels). For these alleles mapped to consensus genomes, the SNP/InDel-index between Lignan-L and Lignan-H bulks was calculated using a sliding window approach with a window size of 1 Mb and a step size of 50 Kb. For example, if both bulks have an identical nucleotide base at an identified position, the SNP/InDel-index value at this position will be 0. Conversely, if the reference genome and the identified nucleotide base are completely inconsistent, the SNP/InDel-index value will be 1. Following the formula: the △(SNP/InDel-index) value as the subtraction of the index between Lignan-L and Lignan-H was plotted as a mirror-like reflected image across all 13 chromosomes according to the two parental consensus genomes (Supplementary Figure S2).

**FIGURE 2:**
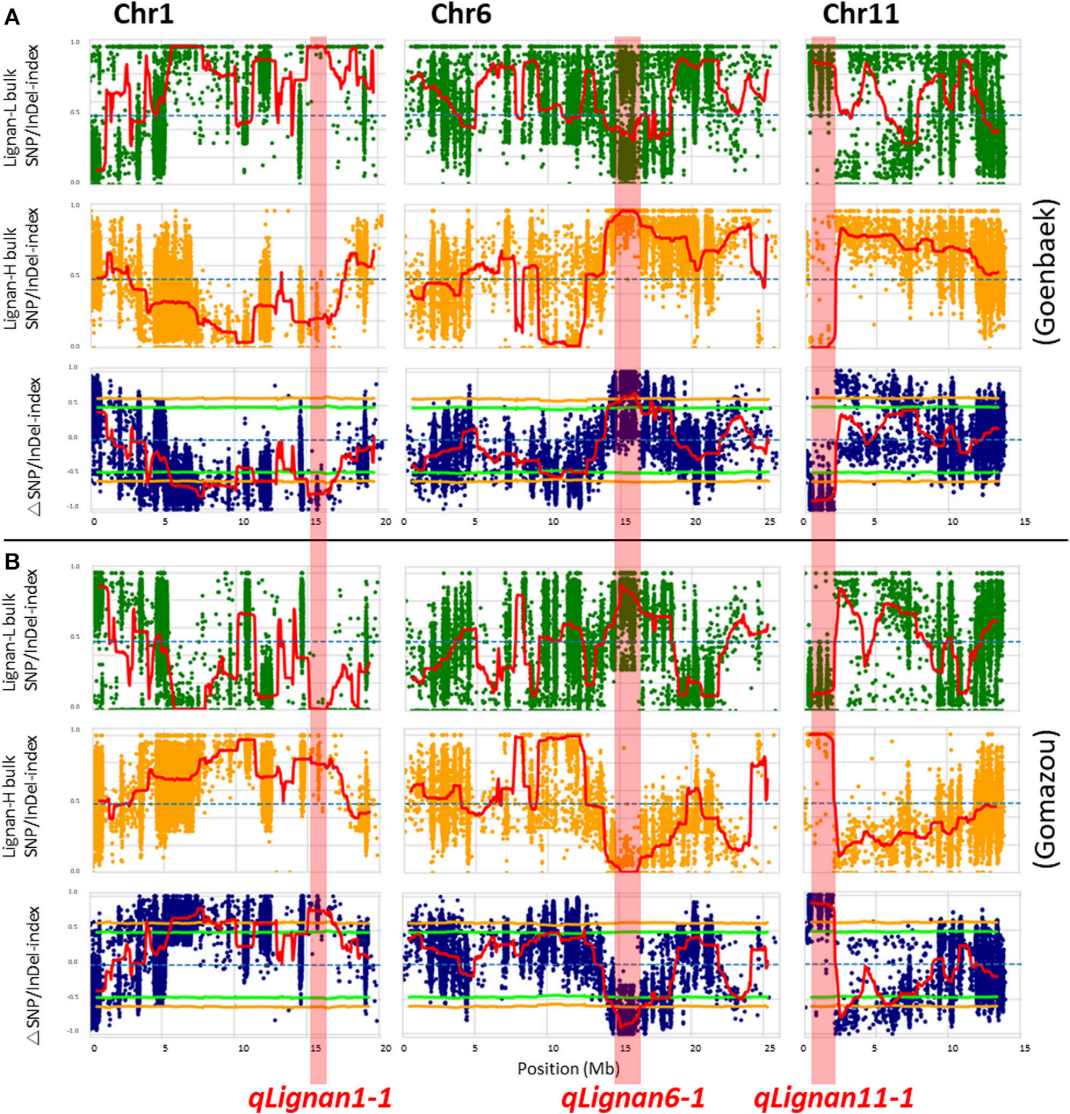
QTL-seq analysis for the identification of major effect QTLs controlling lignan content in sesame. **(A)** SNP/Indel-index plot for high- (green) and low-lignan (yellow) bulks, and △SNP/InDel-index (blue) based on ‘Goenbaek’ as a reference. **(B)** SNP/Indel-index plots for high- (green) and low-lignan (yellow) bulks, and △SNP/InDel-index (blue) based on ‘Gomazou’ as a reference. The average index value was plotted using red lines using a sliding window approach of 1 Mb intervals with 50 kb increments. The △SNP/InDel-index obtained by subtracting the SNP/InDel-index of low-lignan bulk from the SNP/InDel-index of high-lignan bulk was calculated with the statistical confidence interval under the null hypothesis of no QTL (orange, *p* < 0.01; and light green, *p* < 0.05). The three major effect QTLs with the |△SNP/InDel-index|≥ 0.7 were shadowed in red on chromosome 1, 6, and 11, and designated as *qLignan1-1*, *qLignan6-1*, and *qLignan11-1*, respectively.

After calculating the △(SNP/InDel-index) value using Goenbaek and Gomazou as references, 12 and 13 genomic regions were obtained as candidate QTLs controlling lignin at a 99% significance level, respectively, and a total of 10 common genetic regions were found (Table 2). Among them, three major QTLs with △(SNP/InDel-index) > 0.7, which were expected to be closely associated with the trait, were identified on chromosomes 1, 6, and 11, and were namely: *qLignan1-1*, *qLignan6-1*, and *qLignan11-1* (Figure 2). The *qLignan6-1* region comprises 1.9 Mb (14,600,000–16,500,000 bp), and the △(SNP/InDel-index) value was close to ‘1’ when calculated on the basis of Goenbaek and close to ‘-1’ when calculated on the basis of Gomazou (Table 2; Figure 2). This result implies that this QTL region consists of a pool (Lignan-H) of individuals with a region inherited from high lignin in Gomazou and a pool (Lignan-L) of individuals with a region inherited from low lignin in Goenbaek, and lignan content is partially regulated by underlying genetic factors in this region. In contrast, *qLignan1-1* and *qLignan11-1* comprise a region of 1.65 Mb (15,100,000–16,750,000 bp) and 1.6 Mb (500,000–2,100,00 bp), respectively, and showed the opposite pattern of index values to *qLignan6-1* (Table 2; Figure 2).

**TABLE 2:** Number of significant loci and SNP/InDel from QTL-seq analysis.

Consensus genome	Chromosome	Confidence interval (bp)	SNP/InDel index		△(SNP/InDel-index)
			Lignan-L	Lignan-H	
Goenbaek	chr1	3,950,000	0.9305	0.284	−0.6465
		5,650,000–10,100,000	0.9044	0.2155	−0.6889
		11,400,000	0.8871	0.2597	−0.6273
		11,600,000–11,750,000	0.9123	0.3052	−0.6071
		12,850,000–12,900,000	0.8936	0.264	−0.6285
		15,100,000–16,750,000	0.9774	0.2078	−0.7696
	chr2	14,850,000–15,250,000	0.8667	0.2131	−0.6536
	chr3	23,150,000–23,600,000	0.9971	0.3881	−0.609
	chr6	15,100,000–16,050,000	0.3565	0.9978	0.6414
	chr9	6,000,000–6,350,000	0.9549	0.3348	−0.6201
		6,550,000–6,700,000	0.9558	0.3378	−0.6181
	chr11	500,000–2,100,000	0.8743	0.0206	−0.8537
Gomazou	chr1	3,950,000	0.081	0.7174	0.6364
		5,650,000–10,100,000	0.0992	0.7779	0.6787
		11,400,000	0.1228	0.7369	0.6142
		11,600,000	0.088	0.6968	0.6087
		12,850,000–12,900,000	0.1111	0.7391	0.628
		15,100,000–16,750,000	0.0243	0.7923	0.768
	chr2	14,950,000–15,250,000	0.1358	0.7853	0.6495
	chr3	23,150,000–23,600,000	0.003	0.6196	0.6166
	chr6	14,600,000–16,500,000	0.8129	0.021	−0.792
	chr11	500,000–2,100,000	0.1277	0.99	0.8623
		2,450,000–3,150,000	0.8427	0.1523	−0.6904
	chr12	14,350,000–15,750,000	0.7222	0.0054	−0.7167
	chr13	2,550,000–2,600,000	0.2645	0.869	0.6045

### 3.4 Functional classification of variants and candidate gene search

**FIGURE 3:**
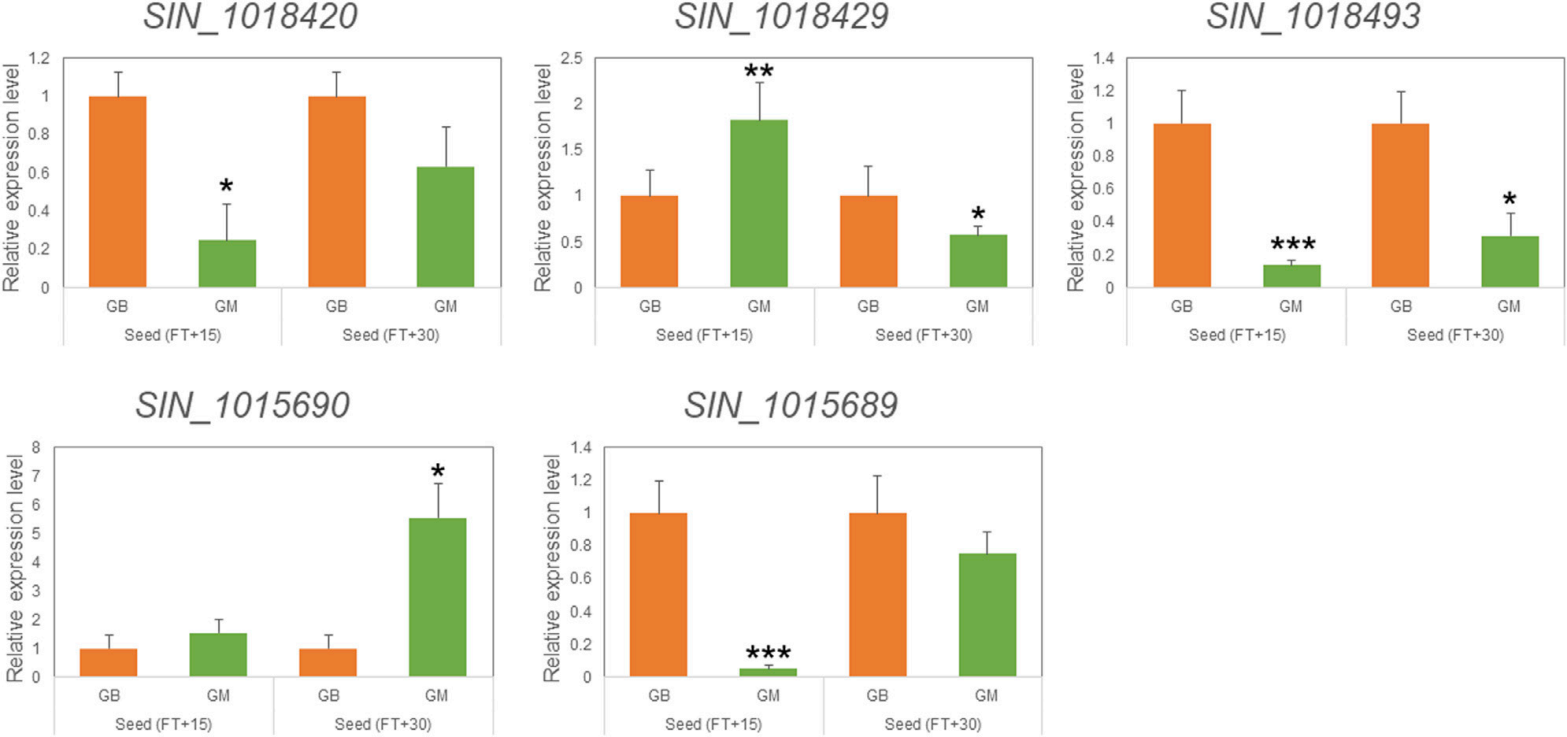
qRT-PCR analysis of selected genes mapped in the KEGG pathway for the discovery of candidate genes. Relative expression of five genes in seeds at 15 and 30 days after flowering (FT) between Goenbaek (GB, orange) and Gomazou (GM, green). Error bars represent standard errors of three biological replicates. Statistically significant differences are indicated as a *p*-value <0.05, *, <0.01, **, and <0.001, ***.

Variant analysis was performed to explore candidate SNPs/InDels or genes associated with sesame seed lignin content. First, we used the information from the reference genome to search for genes located in three QTL intervals, *qLignan1-1*, *qLignan6-1*, and *qLignan11-1*, and found 220, 195, and 146 genes, respectively (Table 3). A total of 2,272 variants were then selected, with counter-SNPs/InDels being defined as variants that were completely biased to one side between the bulks representing index values of 1 or -1. Using their location, the functional classification of the variant was performed according to the annotation and expected change in function within or near the gene. As a result, a total of 11 counter-SNPs/InDels in *qLignan1-1* were found to be in the intron region of one gene, 468 and 232 counter-SNPs/InDels in *qLignan6-1* and *qLignan11-1*, respectively, were associated with 60 and 18 genes, respectively (Table 3). To find genes that were previously reported to affect secondary metabolite synthesis, functional descriptions of the genes were obtained using BLASTP against *A. thaliana* protein sequences. The KEGG analysis revealed that out of a total of 73 genes, 18 were assigned KEGG orthology accession numbers and mapped to 34 pathways, with 10 genes belonging to metabolic pathways, followed by seven genes belonging to secondary metabolite biosynthesis (Table 4). Based on the genes assigned to metabolic pathways, five genes were selected for transcriptional expression analysis (Figure 3). Among them, three genes (*SIN_1018420*, *SIN_1018429*, *SIN_1018493*) have CDS variants with altered amino acid sequences, and two genes (*SIN_1015690* and *SIN_1015689*) belonging to the tyrosine and phenylalanine pathway. The results revealed significantly lower levels of *SIN_1018420*, a homolog of *AT1G12240* (glycosylhydrolase family 32 protein), in immature seeds at the early stage (15 days after flowering) of Gomazou compared to those of Goenbaek. At both maturation stages, significant differences in gene expression were observed between the parental lines for *SIN_1018429*, a homolog of *AT2G30575* (adenine phosphoribosyltransferase 4), and *SIN_1018493*, a homolog of *AT2G30575* (los glycosyltransferase 5). Two gene homologs of *AT4G12290*, *SIN_1015690*, and *SIN_1015689*, which encode proteins of the copper amine oxidase family, exhibited different patterns of gene expression. Specifically, *SIN_1015690* showed higher expression in the Gomazou compared to the Goenbaek during later stages of immature seeds (30 days after flowering). Conversely, *SIN_1015689* showed significantly lower gene expression in the Gomazou than in the Goenbaek during early stages of immature seeds.

**TABLE 3:** Summary of the number of counter-SNPs/InDels near/or within genes in QTL regions.

QTLs (genes)		Number of counter-SNPs/InDels (genes)													
		1 kb upstream		5′UTR		CDS		Intron		3′UTR		1 kb downstream		Total	
*qLignan1-1*	(220)	0	(0)	0	(0)	0	(0)	11	(1)	0	(0)	0	(0)	11	(1)
*qLignan6-1*	(195)	125	(35)	0	(0)	62	(25)	141	(25)	0	(0)	140	(44)	468	(60)
*qLignan11-1*	(146)	74	(14)	0	(0)	31	(9)	76	(15)	0	(0)	51	(11)	232	(18)

**TABLE 4:** Lists of 79 genes functionally annotated in three QTLs and functional description based on *Arabidopsis thaliana* homologues with KEGG orthology.

Gene ID	1 kb upstream	CDS				Intron	1 kb downstream	A.thaliana gene ID	Gene description^a^	KO^b^
		Synonymous	Missense	Nonsense	Frameshift					
SIN_1023526	0	0	0	0	0	11	0	AT2G41830	Uncharacterized protein	
SIN_1015666	0	0	1	0	0	0	0	AT2G14095	unknown protein	
SIN_1015667	0	0	0	0	0	1	0	AT5G55230	microtubule-associated proteins 65-1	
SIN_1015668	0	3	1	0	0	1	0	AT1G12420	ACT domain repeat 8 (ACR8)	
SIN_1015669	0	0	0	0	0	0	6	AT4G12080	AT-hook motif nuclear-localized protein 1 (AHL1)	
SIN_1015671	1	0	0	0	0	0	3	AT1G12410	CLP protease proteolytic subunit 2	
SIN_1015672	0	0	1	0	0	0	2	AT3G05100	S-adenosyl-L-methionine-dependent methyltransferases superfamily protein	
SIN_1015673	0	0	0	0	0	0	1	AT4G22790	MATE efflux family protein	
SIN_1015674	0	1	0	0	0	0	0	AT1G04110	Subtilase family protein	
SIN_1015676	2	0	0	0	0	0	1	AT2G27830	unknown protein	
SIN_1015679	1	0	0	0	0	0	0	AT1G12370	photolyase 1 (PHR1)	
SIN_1015680	0	0	0	0	0	1	1	AT1G12360	Sec1/munc18-like (SM) proteins superfamily	
SIN_1015683	0	0	0	0	0	2	0	AT4G22740	glycine-rich protein	
SIN_1015687	0	2	0	0	0	10	0	AT4G12230	alpha/beta-Hydrolases superfamily protein	K19367
SIN_1015688	0	0	0	0	0	0	1	AT1G02000	UDP-D-glucuronate 4-epimerase 2 (GAE2)	K08679
SIN_1015689	1	0	0	0	0	16	0	AT4G12290	Copper amine oxidase family protein	K00276
SIN_1015690	1	1	0	0	0	4	0	AT4G12290	Copper amine oxidase family protein	K00276
SIN_1015691	2	0	2	0	0	0	0	AT4G12300	polypeptide 4 (CYP706A4)	
SIN_1015692	0	0	0	0	0	7	0	AT4G12320	polypeptide 6 (CYP706A6)	
SIN_1015693	0	0	3	0	0	8	3	AT2G30370	CHALLAH (CHAL)	
SIN_1018396	6	0	0	0	0	0	2	AT4G12300	polypeptide 4 (CYP706A4)	
SIN_1018406	0	0	0	0	0	0	2	AT2G26900	Sodium Bile acid symporter family	
SIN_1018407	9	1	0	0	0	11	1	AT4G12350	myb domain protein 42 (MYB42)	
SIN_1018408	3	2	1	1	0	0	2	AT3G24255	RNA-directed DNA polymerase (reverse transcriptase)-related family protein	
SIN_1018409	2	0	0	0	0	0	7	AT5G64880	unknown protein	
SIN_1018410	3	0	0	0	0	4	2	AT1G62780	unknown protein	
SIN_1018411	9	0	0	0	0	0	1	AT4G12390	pectin methylesterase inhibitor 1 (PME1)	
SIN_1018412	2	0	0	0	0	0	0	AT5G62360	Plant invertase/pectin methylesterase inhibitor superfamily protein	
SIN_1018413	10	2	0	0	0	1	2	AT1G62750	Translation elongation factor EFG/EF2 protein	
SIN_1018414	3	0	0	0	0	2	1	AT3G10195	Putative membrane lipoprotein	
SIN_1018415	2	0	0	0	0	1	1	AT4G12400	stress-inducible protein, putative	
SIN_1018416	0	0	0	0	0	0	10	AT4G22620	SAUR-like auxin-responsive protein family	K14488
SIN_1018417	0	0	0	0	0	12	7	AT1G12250	Pentapeptide repeat-containing protein	
SIN_1018420	0	5	1	0	0	4	0	AT1G12240	Glycosyl hydrolases family 32 protein	K01193
SIN_1018421	1	0	0	0	0	5	5	AT1G12230	Aldolase superfamily protein	K00616
SIN_1018422	1	1	0	0	0	1	5	AT4G22590	Haloacid dehalogenase-like hydrolase (HAD) superfamily protein	K01087
SIN_1018423	2	0	0	0	0	0	2	AT5G55550	RNA-binding (RRM/RBD/RNP motifs) family protein	K14411
SIN_1018424	0	0	0	0	0	0	4	AT4G22600	unknown protein	
SIN_1018425	2	0	0	0	0	0	6	AT4G22580	Exostosin family protein	
SIN_1018426	6	0	3	0	0	0	2	AT1G63370	Flavin-binding monooxygenase family protein	
SIN_1018427	0	0	1	0	0	5	0	AT1G63370	Flavin-binding monooxygenase family protein	
SIN_1018428	1	0	0	0	0	0	4	AT1G62530	Plant protein of unknown function (DUF863)	
SIN_1018429	6	0	1	0	0	0	2	AT4G12440	adenine phosphoribosyl transferase 4 (APT4)	K00759
SIN_1018430	5	0	0	0	0	0	2	AT1G62520	unknown protein	
SIN_1018431	7	0	1	0	0	0	2	AT5G49500	Signal recognition particle, SRP54 subunit protein	K03106
SIN_1018432	2	3	0	0	0	8	2	AT4G22540	OSBP(oxysterol binding protein)-related protein 2A (ORP2A)	
SIN_1018433	8	2	1	0	0	3	2	AT1G12110	nitrate transporter 1	
SIN_1018434	0	0	0	0	0	6	0	AT3G12810	SNF2 domain-containing protein/helicase domain-containing protein	K11661
SIN_1018435	7	0	0	0	0	0	11	AT1G12100	Bifunctional inhibitor/lipid-transfer protein/seed storage 2S albumin superfamily protein	
SIN_1018437	2	0	0	0	0	0	6	AT1G12100	Bifunctional inhibitor/lipid-transfer protein/seed storage 2S albumin superfamily protein	
SIN_1018438	2	0	0	0	0	5	3	AT2G13620	cation/hydrogen exchanger 15 (CHX15)	
SIN_1018440	1	5	4	0	0	0	3	AT4G15550	indole-3-acetate beta-D-glucosyltransferase (IAGLU)	
SIN_1018441	0	1	0	0	0	0	6	AT4G12490	Bifunctional inhibitor/lipid-transfer protein/seed storage 2S albumin superfamily protein	
SIN_1018442	2	0	0	0	0	0	3	AT2G45180	Bifunctional inhibitor/lipid-transfer protein/seed storage 2S albumin superfamily protein	
SIN_1018443	0	0	0	0	0	0	1	AT2G45180	Bifunctional inhibitor/lipid-transfer protein/seed storage 2S albumin superfamily protein	
SIN_1018444	5	0	5	1	0	0	3	AT3G26120	terminal EAR1-like 1	
SIN_1018446	1	1	0	0	0	1	7	AT4G17260	Lactate/malate dehydrogenase family protein	K00016
SIN_1018447	0	2	1	0	0	22	1	AT4G16510	YbaK/aminoacyl-tRNA synthetase-associated domain	
SIN_1018448	7	0	0	0	0	0	1	AT4G16515	root meristem growth factor 6 (RGF6)	
SIN_1018492	0	0	0	0	0	0	1	AT4G16600	Nucleotide-diphospho-sugar transferases superfamily protein	
SIN_1018493	0	0	0	0	1	0	0	AT2G30575	los glycosyltransferase 5 (LGT5)	K13648
SIN_1005674	0	0	0	0	0	1	0	AT1G16760	Protein kinase protein with adenine nucleotide alpha hydrolases-like domain	
SIN_1005676	1	0	0	0	0	0	1	AT4G01250	WRKY family transcription factor22	K13425
SIN_1005696	1	0	0	0	0	0	0	AT4G18020	CheY-like two-component responsive regulator family protein	
SIN_1009908	2	0	0	0	0	14	0	AT2G45650	AGAMOUS-like 6 (AGL6)	
SIN_1009909	0	3	4	0	0	4	21	AT4G00230	xylem serine peptidase 1 (XSP1)	
SIN_1009910	0	3	0	0	1	4	0	AT4G00230	xylem serine peptidase 1 (XSP1)	
SIN_1009911	21	0	0	0	0	6	0	AT1G01910	P-loop containing nucleoside triphosphate hydrolases superfamily protein	
SIN_1009951	1	0	0	0	0	1	1	AT3G53740	Ribosomal protein L36e family protein	K02920
SIN_1009952	3	2	0	0	0	12	0	AT2G45880	beta-amylase 7	K01177
SIN_1009953	2	4	7	0	0	5	12	AT4G00440	Protein of unknown function (DUF3741)	
SIN_1009954	21	0	0	0	0	2	1	AT1G01650	SIGNAL PEPTIDE PEPTIDASE-LIKE 4 (SPPL4)	
SIN_1009955	0	1	0	0	0	6	1	AT3G61415	SKP1-like 21 (SK21)	
SIN_1009956	6	0	0	0	0	0	0	AT1G01610	glycerol-3-phosphate acyltransferase 4 (GPAT4)	K13508
SIN_1009957	4	0	0	0	0	3	3	AT4G00370	Major facilitator superfamily protein	
SIN_1009958	2	0	1	0	0	1	1	AT2G46020	transcription regulatory protein SNF2, putative	
SIN_1009959	1	1	0	0	0	8	1	AT1G63800	ubiquitin-conjugating enzyme 5 (UBC5)	K10576
SIN_1009960	2	1	1	0	0	2	6	AT4G00335	RING-H2 finger B1A (RHB1A)	
SIN_1009961	7	1	1	0	0	7	3	AT1G01580	ferric reduction oxidase 2	

^a^ The description follows *Arabidopsis thaliana* genes of TAIR, version10.

^b^ KO: Kyoto Encyclopedia of Genes and Genomes (KEGG) orthology.

### 3.5 Additive effect of alleles and QTL stacking

**FIGURE 4:**
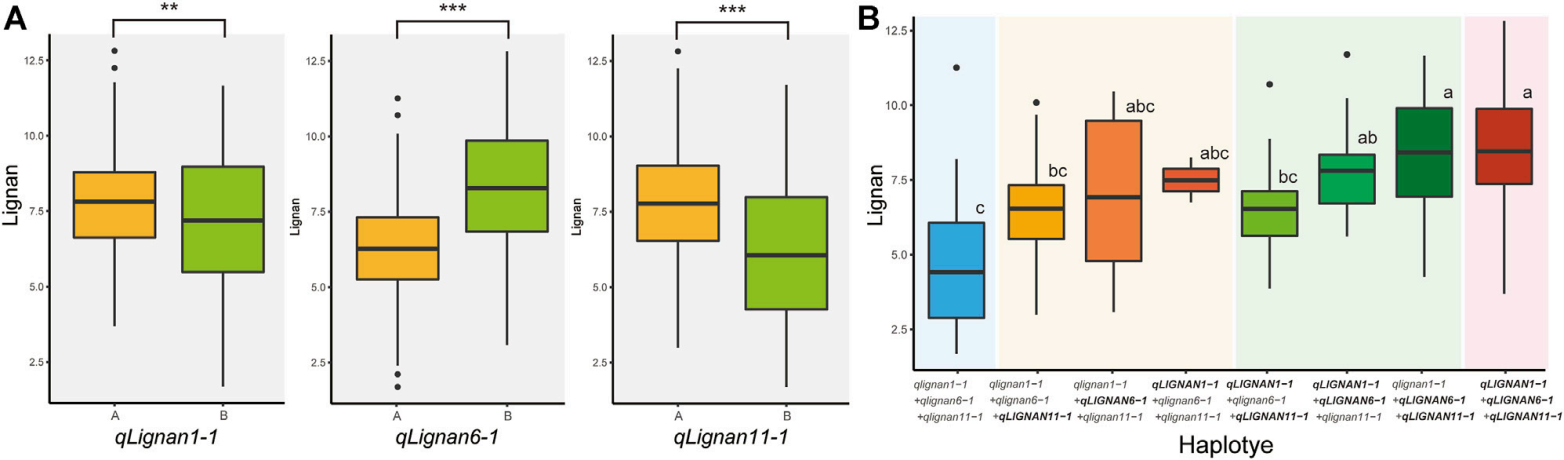
Comparison of lignan content effects across alleles between three QTLs. **(A)** Results of the effect of the lignan content of individual alleles. Genotype ‘A’ refers to alleles derived from the parent, Goenbaek, and ‘B’ refers to alleles derived from Gomazou. Statistically significant differences are indicated as a *p*-value <0.01, **, and <0.001, ***. **(B)** Results of the effect of the lignan content by combinations of alleles. Each QTL designation denotes alleles in the group with higher lignan content as large. Post hoc tests were used to indicate statistical significance.

To investigate the allelic effect of the three QTL with high index values and to correlate the genotype and phenotype of individuals in the population, KASP, InDel, and CAPS markers were developed using counter-SNP/InDel (Table 5). The allelic genotyping markers were used for variants located in the CDS region of *qLignan6-1*, in the intergenic region of *qLignan11-1*, and in the intron region of *qLignan1-1*. The differences in lignan content associated with each QTL allele in the population were statistically significant (Figure 4A). Specifically, *qLignan1-1* and *qLignan11-1* exhibited higher lignan content along the Goenbaek allele, while *qLignan6-1* showed higher lignan content along the Gomazou allele. The investigation of potential interactions among the QTLs revealed that specific combinations of alleles resulted in high-lignan content. Combinations of two or more alleles were found to contribute to an increase in lignan content in comparison to single alleles. The highest lignan content was observed when all three QTL alleles were incorporated (Figure 4B). Combinations including *Lignan6-1*, which showed significantly higher lignan content when combined with other QTLs, were particularly noticeable. This finding provides compelling evidence for the substantial contribution of the Gomazou-derived allele to lignan content.

**TABLE 5:** DNA marker information to identify alleles for lignan content controlling in sesame.

Marker	Position	Goenbaek	Gomazou	Primer sequence
InDel	Si1_15740491	TTT​AAT​TTA​TTT​CAT​TAA​TTT​A	TTTAATTTA	F: TCG​TAC​GCA​TGA​GAT​GAA​ACA R: CAT​CCC​AAA​TCC​CAA​GTT​GT
KASP	Si6_15239077	A	G	Primer allele X
				CCT​TCA​CCA​ATT​CCC​TCC​TCG
				Primer allele Y
				CCC​TTC​ACC​AAT​TCC​CTC​CTC​A
				Primer common
				AAC​TGA​AAG​GCT​CAA​GCG​GCG​TTT
CAPS (*BspHI*)	Si11_833990	T	C	F: ATT​CGA​TCG​ATG​CCC​CAT​AG
				R: TTC​ATG​TAA​CGA​GGA​ATC​AAT​CA

## 4 Discussion

Identification of QTLs and the development of molecular markers that are closely linked to useful genes can be a useful tool in the breeding of new varieties by applying MAS, saving time and labor, and accelerating breeding cycles (Paran and Zamir, 2003; Collard et al., 2005). It is important that QTLs or markers are used to estimate phenotypic expression at the molecular level, particularly because direct measurement of quantitative traits is difficult (Nadeem et al., 2018). The SNP markers associated with the *ahFAD2* gene, responsible for the introgression of high oleic acid content in peanut and SSR markers for the selection of the *Gpc-B1* gene, responsible for the selection of high grain protein varieties, are examples of advancing the effectiveness of breeding using marker selection resulting from QTL studies (Vishwakarma et al., 2014; Bera et al., 2018). Recently, by fusing genome-level information on components such as secondary metabolites with information on the biosynthesis of compounds using advanced NGS technology, research is expanding into a new area called the metabolome (Keurentjes, 2009; Carreno-Quintero et al., 2013). For example, 4,681 metabolic quantitative trait loci (mQTLs) were identified as profiling metabolites that accumulate in seeds at different developmental stages in rice (Li et al., 2019), and 42 loci were identified from 1,098 associations in a metabolite-based genome-wide association study (mGWAS), thus suggesting breeding strategies for flavonoid pathway metabolites in wheat (Chen et al., 2020).

Finding a resource that is suitable for the target trait and generating a genetic population to find the genes that control the trait are necessary to achieve successful QTL results. In this study, to find the regulators of lignan content in sesame, an F7 RIL population was established using Goenbaek, an elite variety with stable cultivation but low-lignan content, and Gomazou, a variety with high-lignan content, as parental lines. We then compared sequence information from a pool of transgressive segregating individuals containing high and low-lignan content for the QTL analysis. Based on overlapped genetic regions from comparing two parental versions as a reference for the calculation of sequence frequencies to avoid errors caused by heterozygous or missing positions in the mapping process, three major QTLs (*qLignan1-1*, *qLignan6-1*, and *qLignan11-1*) were identified. In previous studies, QTL results using the highly biased variant frequency index in separated bulks have been shown to be reliable in comparison to traditional QTL analysis in rice, tomato, and maize (Chen et al., 2021; Topcu et al., 2021; Yang et al., 2021). The identified *qLignan11-1* is also located in close proximity to *qSim_11.1* and *qSmol_11.1*, which were reported to have high phenotypic effects (67.69% and 46.05%, respectively) on sesamin and sesamolin content using F8 RILs in sesame (Xu et al., 2021). Although using the lignan with the combined content of sesamin and sesamolin as an indicator is a potential limitation of this study, the strong correlation between the components (Supplementary Figure S1) supports our finding on a comprehensive locus that can be used for breeding. However, a genetic approach to the synthesis of sesamin and sesamolin is needed for further functional analysis.

The relatively large size of the QTL region, which ranged from 1.6 to 1.9 Mb and contained 140–220 genes, limited the selection of specific candidate genes. To overcome this limitation, our study followed a two-step approach to candidate gene selection. First, some of the variants were extremely segregated within the region confined to the QTL region, whereas the SNP index values in most of the regions were heterozygous. These extreme variants were defined as counter-SNPs/InDels, with 11, 468, and 232 variants within each QTL region, spanning one, 60, and 18 genes, respectively (Table 3). Then, we conducted a comparative analysis based on the gene annotation of *A. thaliana.* By selecting genes belonging to secondary metabolic pathways, which are expected to be functionally altered due to variations in genomic regions affecting protein coding (Table 4), we validated their expression levels using parental lines with large differences in lignan content. As a result, All five genes, *SIN_1018420*, *SIN_1018429*, *SIN_1018493*, *SIN_1015690,* and *SIN_1015689*, were differentially expressed in the seeds (Figure 3), but further functional studies are needed to elucidate the trait-associated roles of these genes. Our results suggest that despite the large number of genes distributed among the potential loci, the gene and pathway databases can be helpful in the logical selection of promising candidate genes.

Quantitative traits are determined by multiple genes, each of which may have a large or small effect. Since the quantitative trait is often determined by the interaction of multiple genes with environmental influences, it is preferable to use methods such as QTL-seq to find useful genetic regions focusing on major QTL; although there are limitations in detecting minor QTL (Dong et al., 2021; Sheng et al., 2021). While the elucidation of gene functions at the molecular level is important, it is also important that QTL study results are practically applied in breeding through the use of major gene-induced markers for the efficient selection of pedigrees for time and labor reduction (Xu et al., 2018; Yang et al., 2019). This study verified the reliability of the QTL with KASP, InDel, and CAPS markers, showing that the allele derived from the lignan-rich Gomazou, *qLignan6-1*, and two alleles derived from Goenbaek, *qLignan1-1* and *qLignan11-1*, are dominant. The influence of the allele derived from the high-lignan Gomazou is higher compared to the two QTLs derived from the low-lignan Goenbaek, suggesting that the QTLs derived from the Goenbaek are likely to be minor QTLs. Interactions between alleles were also analyzed to exploit the additive allele effect and found that the dominant form of *qLignan6-1* had greater synergy when combined with other QTLs. It is therefore expected that *qLignan6-1* will play an important role in controlling lignan content, in addition to additive effects with other loci. The discovery of *qLignan6-1* will be useful in the breeding process as a marker for the introgression of useful genes from high-lignan resources into a new variety.

Furthermore, lignans in sesame have been shown to be functional components with antioxidant, anti-dementia, and antihypertensive activities, and the genetic mechanisms required for lignan synthesis have been reported using QTL, GWAS, and transcriptomics in several studies (Harada et al., 2020; Xu et al., 2021; Dossou et al., 2023). CYP92B14, an enzyme that converts sesamin to sesamolin and sesaminol, which was elucidated through functional characterization (Harada et al., 2020), and the *SiSNT1* gene located on chromosome 11, inferred through genetic mapping (Xu et al., 2021; Dossou et al., 2023), may explain the genetic mechanisms of lignan synthesis in sesame. However, these are still poorly understood compared to other major crops. In this study, we found two novel genetic contributors that regulate lignan content, *qLignan1-1* and q*Lignan6-1*, along with *qLignan11-1* at the same location as in previous study. By showing the utility of marker interaction that can be used in practical breeding strategies, we are confident that this will be useful as a basic resource for the development of high-functional lignan varieties.

## Data Availability

The data presented in the study are deposited in the National Center for Biotechnology Information (NCBI) repository (https://www.ncbi.nlm.nih.gov/) under BioProject accession number PRJNA1028140.
