# Supplementary material for: Identification of QTLs and allelic effect controlling lignan content in sesame (Sesamum indicum L.) using QTL-seq approach
Source: Front Genet. 2023 Dec 11;14:1289793. doi: 10.3389/fgene.2023.1289793 (PMC10750367; doi:10.3389/fgene.2023.1289793)
Supplement: Supplementary file 1 [file Table1.DOCX]

Supplementary Material

Identification of QTLs and allelic effect controlling lignan content in sesame (Sesamum indicum L.) using QTL-seq approach

**Sungup Kim^1†^, Eunsoo Lee^1†^, Jeongeun Lee^1^, Yeon Ju An^1^, Eunyoung Oh^1^, Jung In Kim^1^, Sang Woo Kim^1^, Min Young Kim^1^, Myoung Hee Lee^1^, Kwang-Soo Cho^2*^**

# Supplementary Figures and Tables

## Supplementary Figures


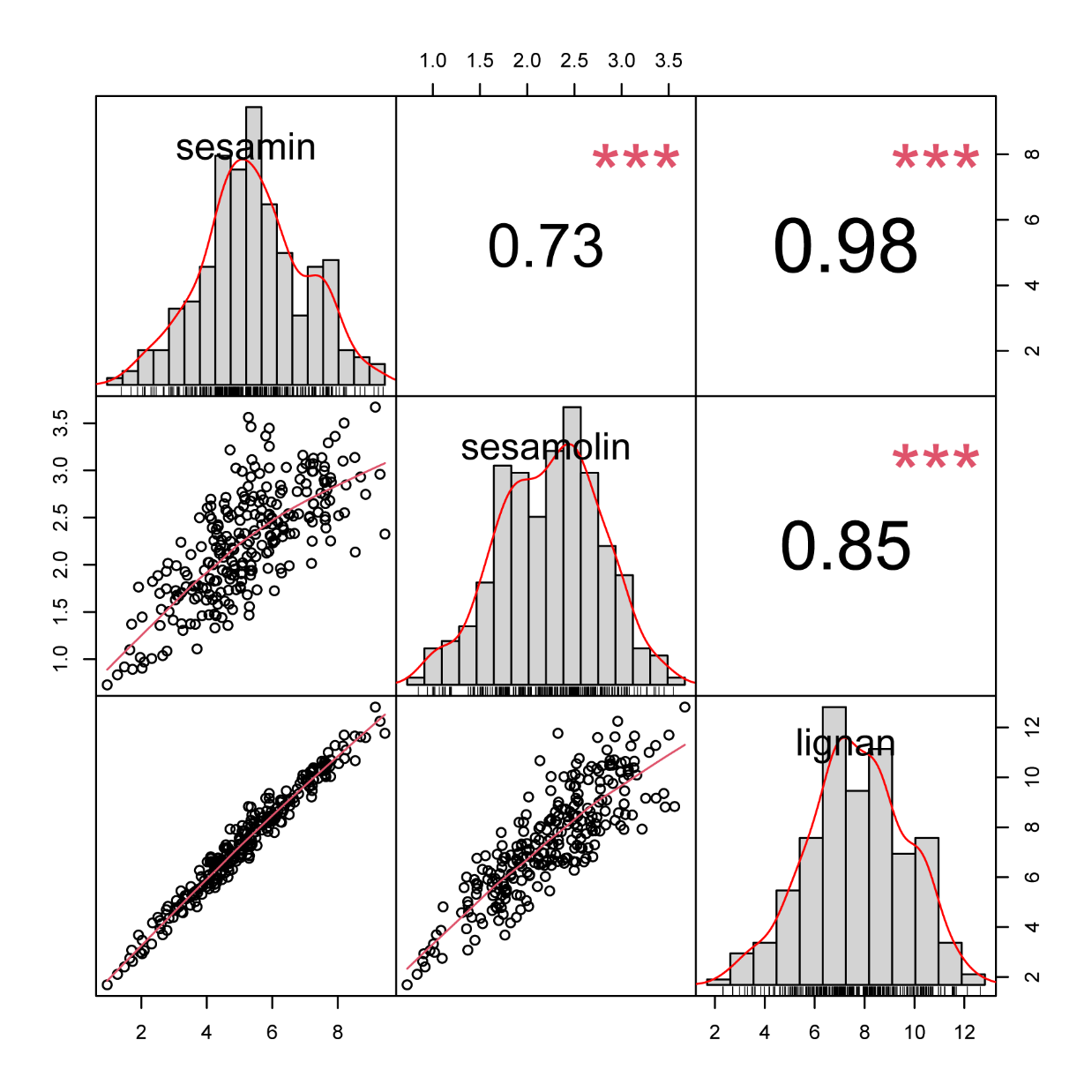


Supplementary Figure S1. Distribution of the content of sesamin, sesamolin, and lignan in the RILs derived from Goenback and Gomazou and their correlations. Statistical significance of Pearson correlation: *** *P* < 0.001.


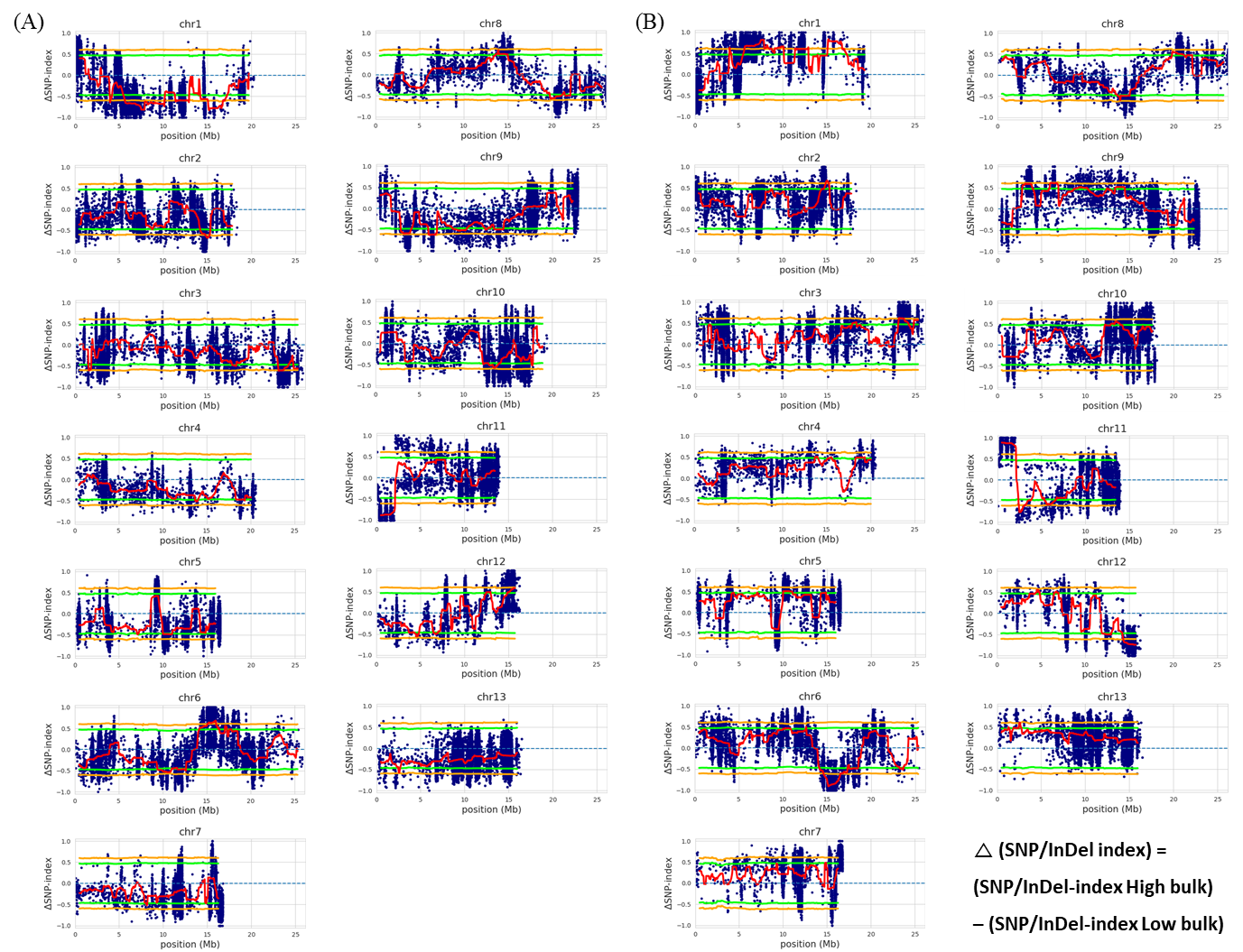


Supplementary Figure S2. △(SNP/InDel-index) plot graph of all 13 chromosomes from the QTL-seq for identifying genomic regions controlling lingnan content in sesame. (A) △SNP/InDel-index plot with ‘Gonbaek’ as a reference. (B) △SNP/InDel-index plot with ‘YCS71’ as a reference. The red line is plotted by average △(SNP/InDel-index) using a sliding window size of 1 Mb intervals with a step of 50 Kb. All chromosome positions in the genome are calculated with the statistical confidence interval under the null hypothesis of no QTL (orange, P<0.01; and green, P<0.05).

## Supplementary Tables

Supplementary Table S1. List of primer information for qRT-PCR analysis.

| Purpose | Gene ID | Primer sequence (5’-3’) |
| --- | --- | --- |
| qRT-PCR | SIN_1018420 | F: GCTCAGCAACCATCCTTCAG  R: GTGGGACCAAGACCGGATTA |
|  | SIN_1018429 | F: TCGCGGCTTTATATTTGGTC  R: AACTACCAAGGCACGCTCAC |
|  | SIN_1018431 | F: CACACATCCACTCCAACAGC  R: TCAAGTTCTGCCCTCTCCAT |
|  | SIN_1018493 | F: AGCAAAGACAAAAGCTGGAGA  R: TGCTAACGGACTTTGGTCTG |
|  | SIN_1015690 | F: TTCTCCAGAAACCCACCAAC  R: GAAGCGCATAGGCTTTGTTC |
|  | SIN_1015689 | F: TCCTGCTCTTCACAGCCTTC  R: TAGACCGATTTCCCGAAGAA |

Supplementary Table S2. Sesamin, sesamolin, and lignan content of selected individuals belonging to extreme bulks for QTL-seq.

| Entry No. | Sesamin (mg/g) | Sesamolin (mg/g) | Lignan (mg/g) | Bulk |
| --- | --- | --- | --- | --- |
| 038149 | 1.0 | 0.7 | 1.7 | Low |
| 038114 | 1.3 | 0.8 | 2.1 | Low |
| 038116 | 1.5 | 0.9 | 2.4 | Low |
| 038113 | 1.7 | 0.9 | 2.6 | Low |
| 038138 | 1.7 | 1.1 | 2.8 | Low |
| 038119 | 2.0 | 0.9 | 2.9 | Low |
| 038100 | 2.0 | 1.0 | 3.0 | Low |
| 038143 | 1.7 | 1.4 | 3.1 | Low |
| 038126 | 2.3 | 1.0 | 3.3 | Low |
| 038108 | 2.0 | 1.5 | 3.5 | Low |
| 038227 | 8.2 | 2.6 | 10.8 | High |
| 038225 | 8.3 | 2.9 | 11.1 | High |
| 038224 | 7.9 | 3.4 | 11.3 | High |
| 038223 | 8.9 | 2.8 | 11.6 | High |
| 038222 | 8.7 | 2.9 | 11.6 | High |
| 038221 | 8.5 | 3.1 | 11.7 | High |
| 038220 | 8.2 | 3.5 | 11.7 | High |
| 038219 | 9.4 | 2.3 | 11.8 | High |
| 038218 | 9.3 | 3.0 | 12.3 | High |
| 038217 | 9.2 | 3.7 | 12.8 | High |

Supplementary Table S3. Summary of resequencing results of parental lines, Lignan-L and Lignan-H, high and low lignan content bulk with ten individual plants, respectively.

| Sample | Total bases | Total reads^a^ | Trimmed reads | Mapped reads | Coverage (%)^b^ | Mapping depth (X)^c^ |
| --- | --- | --- | --- | --- | --- | --- |
| Gonbaek | 24,087,269,416 | 238,487,816 | 223,598,990 | 170,831,013 | 95.58 | 51.18 |
| YSC71 | 40,818,468,282 | 270,320,982 | 258,116,298 | 216,367,131 | 95.93 | 103.02 |
| Lignan-L | 10,664,238,194 | 70,624,094 | 60,306,086 | 48,240,157 | 95.90 | 21.34 |
| Lignan-H | 10,736,236,806 | 71,100,906 | 60,341,236 | 48,403,785 | 95.90 | 21.29 |

^a^The read length of YCS71, Lignan-L and Lignan-H is 151 bp, while the read length of Gonbaek is 101 bp.

^b^Coverage and ^c^Mapping depth were calculated on the proportion and average depth of bases detected at least once in the reference genome (cv. Zhongzhi No.13. version 2.0)
